# The influence of the trajectory of obesity indicators on the age of pubertal onset and pubertal tempo in girls: A longitudinal study in Chongqing, China

**DOI:** 10.3389/fpubh.2023.1025778

**Published:** 2023-02-08

**Authors:** Xuan Xi, Di Wu, Wenyi Wu, Yuanke Zhou, Qin Zhang, Yujie Wang, Hong Wang, Qin Liu

**Affiliations:** ^1^School of Public Health, Research Center for Medicine and Social Development, Chongqing Medical University, Chongqing, China; ^2^College of Medical Informatics, Chongqing Medical University, Chongqing, China

**Keywords:** BMI, waist circumference, waist-to-height ratio, pubertal onset, pubertal tempo

## Abstract

**Objective:**

This study aims to explore the influence of the trajectory of obesity indicators on the onset age of different pubertal development characteristics and pubertal tempo among girls.

**Methods:**

Our longitudinal cohort study recruited 734 girls at baseline in May 2014 from a district of Chongqing and followed them at 6-month intervals. Data were available from baseline to the 14th follow-up with a full record of height, weight, waist circumference (WC), breast development, pubic hair, and armpit hair development, as well as the age of menarche. The Group-Based Trajectory Model (GBTM) was fitted for the optimum trajectory of the body mass index (BMI), WC, and waist-to-height ratio (WHtR) of girls before the pubertal onset and menarche. The ANOVA and multiple linear regression model were conducted to analyze the influence of the trajectory of obesity indicators on the onset age of different pubertal development characteristics and pubertal tempo in girls.

**Results:**

Compared with the healthy (gradual BMI increase) group before pubertal onset, the overweight (persistent BMI increase) group has an earlier onset age of breast development (B: −0.331, 95%CI: −0.515, −0.147) and pubic hair development (B: −0.341, 95%CI: −0.546, −0.136). The B2–B5 development time was shorter in girls in the overweight (persistent BMI increase) group (B: −0.568, 95%CI: −0.831, −0.305) and the obese (rapid BMI increase) group (B: −0.328, 95%CI: −0.524, −0.132). The age of menarche was earlier, and the B2–B5 development time was shorter in girls in the overweight (persistent BMI increase) group than in girls in the healthy (gradual BMI increase) group before menarche (B: −0.276, 95%CI: −0.406, −0.146; B: −0.263, 95%CI: −0.403, −0.123). Girls with high WC (rapid WC increase) before menarche had an earlier age of menarche than normal WC (gradual WC increase) (B: −0.154, 95%CI: −0.301, −0.006), and the B2–B5 development time was shorter in girls in the overweight (gradual WHtR increase) group than in girls in the healthy (persistent WHtR increase) (B: −0.278, 95%CI: −0.529, −0.027) group.

**Conclusion:**

Among girls, overweight and obesity (BMI scale) before pubertal onset can not only influence pubertal onset age but also accelerate B2–B5 pubertal tempo. Overweight (BMI scale) and high WC before menarche also have an impact on the age of menarche. Overweight (WHtR scale) before menarche is significantly associated with B2–B5 pubertal tempo.

## Introduction

Puberty, the transition period from childhood to adulthood, is the critical maturation process of secondary sexual development. In girls, sexual development characteristics include breast development, menarche, pubic hair, armpit hair, and other secondary sexual characteristics and sexual organ development (1). The time model of the emergence of these characteristics in pubertal reproductive development is also known as pubertal timing. Pubertal tempo, which can be used to measure the velocity of the process, is also an important indicator of pubertal development. It requires data from multiple time points and over three times is highly recommended (2).

As an essential period of adolescents' physical and psychological changes, puberty is affected by many factors such as genetics, environment, family, and society. Under the influence of internal and external factors, pubertal timing could be relatively early, timely, and delayed compared with the population of the same gender and age (3). Previous studies showed that childhood obesity has a wide range of health complications, including early puberty, accelerated pubertal tempo, increased cardiovascular risk, disturbances of sex hormones, hepatic and orthopedic problems, and psychosocial comorbidities (4–6). Although researchers have studied the relationship between obesity and pubertal development, the results remained controversial.

Many studies showed that girls with obesity had earlier onset of breast and pubic hair development and menarche (7–9). Most research on girls supported a strong association between the rate of sexual maturity and levels of adolescents' obesity. It has been reported that girls with higher BMI will have earlier menarche after puberty, and it led to shorter puberty as a consequence (10). A cohort study in the United States showed that the speed of pubic hair development was positively correlated with BMI Z-score and body weight, while breast development had no significant correlation with BMI Z-score. The average speed of pubic hair development in girls is slower than that of breast development, and pubic hair develops later than the breast (11).

However, the evidence of the relationship between adolescents' obesity and pubertal tempo in China and Western developing countries is not sufficient. Due to the lack of sample size, complex statistical methods, and incomplete detailed follow-up data on pubertal development, researchers are also limited in inferring the relationship between prepubertal and pubertal obesity and pubertal tempo, resulting in a lack of studies on the impact of obesity on pubertal tempo. Moreover, there are no comprehensive results on the relationship between the trajectory of adolescents' obesity indicators and pubertal timing and pubertal tempo.

Therefore, this study aimed to explore the trajectory of obesity indicators at the different ages of pubertal onset and pubertal tempo in girls. Furthermore, some researchers found that WC and WHtR might be more representative of children's body fat distribution and obesity levels than BMI (12). Hence, we suspect that different obesity indicators might be suitable for distinct pubertal development characteristics and trajectories of girls. To achieve the objective, we used BMI, WC, and WHtR as obesity indicators, Tanner stage B2–B5 and B2-menarche as the indicators of puberty tempo (13), Tanner stage 2 (breast, pubic hair, and armpit hair development) as the marker of puberty onset and menarche as puberty development characteristic in girls in a seven-year cohort study in Chongqing, China.

## Subjects and methods

### Subjects

**Figure 1:**
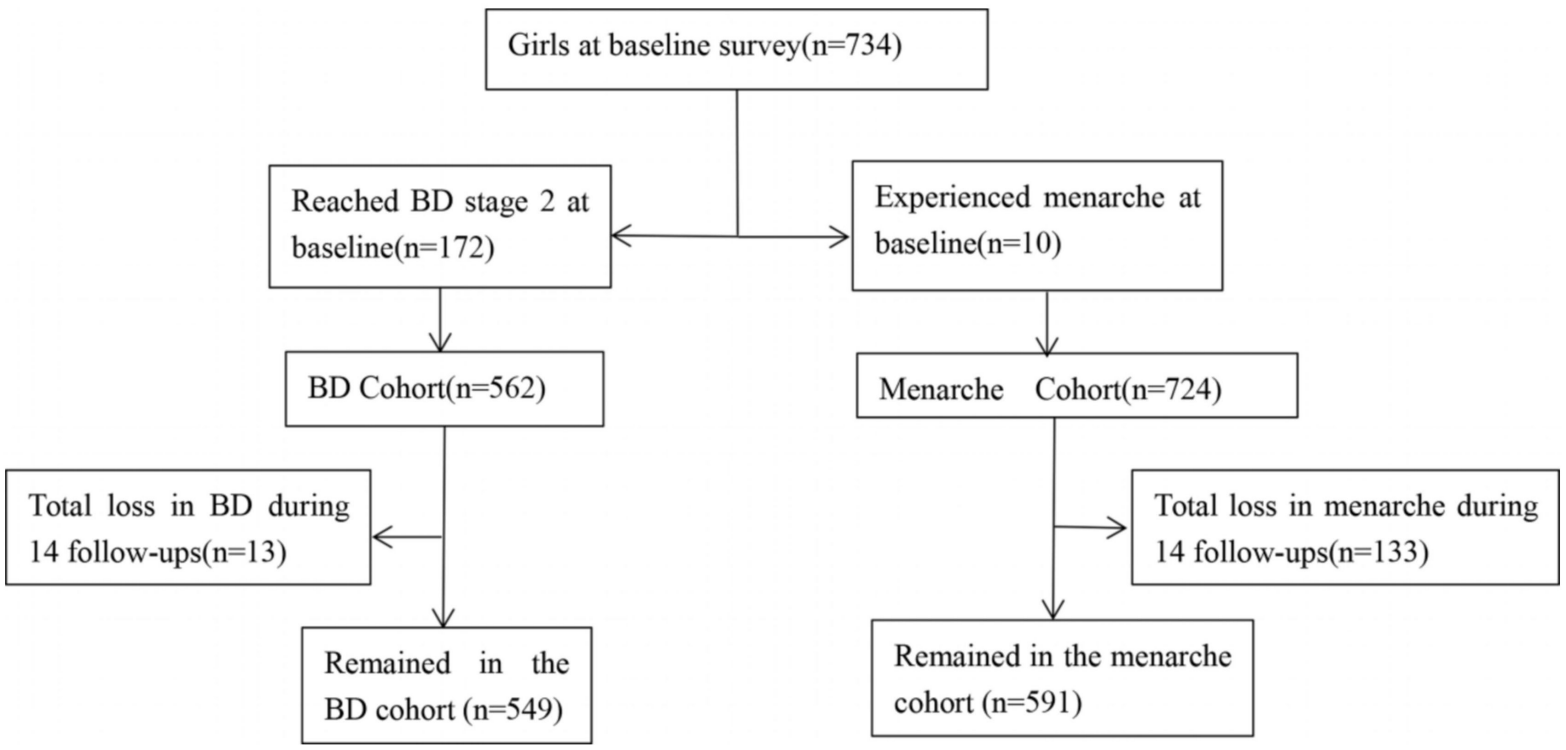
Flowchart of the number of girls with different indicators of pubertal development. BD, Breast development.

The study subjects were from a longitudinal cohort study designed to explore influencing factors of adolescents' pubertal development and its health effects in an urban district of Chongqing, China. In May 2014, a total of 734 girls from grade 1 to grade 4 of four primary schools were recruited at baseline with written consent from students and their guardians and the principle of voluntary confidentiality. Girls were followed up during the 7 years (every 6 months) through in-person examinations and surveys. As of June 2021, 14 follow-ups had been completed (14). Among the 734 girls, we excluded 175 girls who reached breast development stage 2, 10 girls who experienced menarche at baseline, and 133 girls who dropped out during follow-ups. Finally, we included 549 girls whose breast development did not reach stage 2 with an effective follow-up rate of 74.8% and 591 girls who did not have menarche with an effective follow-up rate of 80.5%, see Figure 1. This study was approved by the Medical Ethics Review Committee of Chongqing Medical University.

### Survey tools and variables

The questionnaire contains the basic information variables of adolescents' age, parental relationship (relatively good, general, and relatively bad), parental marital status (married or divorced), parents alive (both alive or one passed away), left-behind children (yes or no), parents' education level (middle school and below, high school or technical secondary school, and college degree or above), and family monthly income.

The physical examination variables included weight, height, and waist circumference. At each examination, weight and height were measured by trained medical graduate students using standard tools. Girls were required to remove their shoes before measuring their height and weight. Standing height was measured with a stadiometer and accurate to 0.1 cm. Weight was measured with a balanced scale and accurate to 0.1 kg. BMI was calculated by height and weight (kg/m^2^). BMI cut-offs for overweight and obesity were based on screening among school-age children and adolescents formulated by the National Health and Family Planning Commission of the People's Republic of China published in 2018 (15). Overweight was defined as BMI greater than or equal to the threshold for overweight and less than the threshold for obesity in the corresponding gender and age group. Obesity was defined as BMI greater than or equal to the obesity threshold of the corresponding gender and age group. WC standard refers to a high WC screening threshold among children and adolescents aged 7–18 years (16). Since age 7 is not included in the WC standard, girls below 7 years old were grouped according to the cut-off value of 85th percentile by age. Girls above the 85th percentile were classified as having a high waist circumference (17). The 75th percentile and 90th percentile of age-specific WC of girls were used as the threshold of normal WC high value and high WC of girls, respectively. WHtR as central obesity screening threshold is 0.5 (18, 19).

Breast, pubic hair, and armpit hair development in girls were assessed by Tanner stages by trained medical graduate students during physical examinations every 6 months (20). Girls were asked to recall the exact menarche time if applicable. Pubertal onset in girls is defined as either experiencing menarche according to self-report or breast development reaching stage 2 at the time of examination. There are also two definitions for the pubertal tempo: (1) the period from Tanner stage 2 to menarche; and (2) the period of breast development from Tanner stage 2 to stage 5.

### Quality control

Before each follow-up, investigators would calibrate the equipment which will be used in the physical examination to ensure the accuracy of data and control system errors. After strict training, all investigators explained the requirements to the students before handing out questionnaires and checked whether any questionnaire information was missing or there were logical errors after questionnaire submission. During the follow-up, investigators tried to establish a good trust relationship with girls to improve compliance. Yet, some girls dropped out during follow-ups as they were entering middle school. The former primary school teachers, students, and local healthcare centers were contacted. Support was obtained from most schools through negotiation, and we continued to carry out follow-up surveys (21, 22).

### Statistical analysis

Epidata3.1 was used to double-enter and validate data. IBM SPSS Statistics 20.0 was used for descriptive statistical analysis, and the chi-square test was used for demographic information and pubertal development data of girls. ANOVA and multiple linear regression were conducted to analyze the influence of the trajectory of obesity indicators on the different ages of pubertal onset and pubertal tempo in girls.

Based on SAS9.3, the Group-Based Trajectory Model (GBTM) was used to model the BMI, WC, and WHtR of girls before the onset ages of puberty and menarche and fitted the optimal trajectory model. GBTM was implemented by the Proc Traj process (23) and included: (1) Select an appropriate analysis model according to the type of research data. In our study, as the obesity indicators including BMI, WC, and WHtR belong to continuous variables, the Censored Normal distribution (CNORM) model was used for fitting. The dependent variable was the data on different obesity indicators, and the independent variable was the age of girls. (2) The fitting effect of the model was determined according to two indicators, Bayesian Information Criterion (BIC) and average posterior probability (AvePP). Meanwhile, relevant professional knowledge and the principle of model simplification were considered. When the BIC value is closer to 0, the model-fitting effect is better. AvePP represented the posterior probability of each individual being divided into the corresponding subgroup, and >0.7 was generally acceptable.

By determining the appropriate number of subgroups, the optimal number and shape of trajectories were finally obtained after repeated fitting. We started fitting from the minimum number of subgroups, and each subgroup was fitted sequentially from high order to low order. If the parameters obtained when selecting high order have no statistical significance, the low-order fitting was selected. According to the actual number of girls included in the analysis and the specific situation, the trajectories of BMI, WC, and WHtR of girls before pubertal onset and menarche were fitted, respectively. Finally, we determined the optimal number of trajectories.

## Results

### Comparison of characteristics and physical examination of enrolled and dropped-out girls before menarche

The mean of BMI, waist circumference, and WHtR was 16.51 ± 2.49, 55.85 ± 7.25, and 0.42 ± 0.05, respectively. The analysis showed that there was a statistically significant difference in parental educational level between enrolled and dropped-out girls (χ^2^:11.669, *p* < 0.01; χ^2^:7.374, *p* < 0.05). No statistically significant differences were found in other indicators (*p* >0.05), see Table 1.

**Table 1:** Comparison of characteristics and physical examination of enrolled and dropped-out girls before menarche.

**Indicators**		**Recruited** **(*n* = 734)**	**Enrolled** **(*n* = 591)**	**Dropped out** **(*n* = 133)**	**t/χ^2^**	***p***
Parents alive	Both alive	724 (98.6)	584 (98.8)	130 (97.7)	0.915	0.339
	One passed away	10 (1.4)	7 (1.2)	3 (2.3)		
Parental relationship	Relatively good	608 (82.8)	491 (83.0)	109 (82.0)	1.621	0.445
	General	69 (9.4)	53 (9.0)	16 (12.0)		
	Relatively bad	57 (7.8)	47 (8.0)	8 (6.0)		
Parental marital status	Divorced	67 (9.1)	56 (9.5)	10 (7.5)	0.502	0.479
	Married	667 (90.9)	535 (90.5)	123 (92.5)		
Left-behind children^a^	Yes	560 (76.3)	461 (78.0)	99 (74.4)	0.789	0.375
	No	174 (23.7)	130 (22.0)	34 (25.6)		
Father education	Middle school and below	337 (45.9)	270 (45.7)	61 (45.9)	11.669	0.003
	High or technical secondary school	236 (32.2)	204 (34.5)	30 (22.6)		
	College degree or above	161 (21.9)	117 (19.8)	42 (31.6)		
Mother education	Middle school and below	360 (49.0)	294 (49.7)	60 (45.1)	7.374	0.025
	High or technical secondary school	232 (31.6)	193 (32.7)	36 (27.1)		
	College degree or above	142 (19.3)	104 (17.6)	37 (27.8)		
Average monthly household income (RMB)	< 2000	228 (31.1)	177 (29.9)	48 (36.1)	1.919	0.383
	2001–4000	329 (44.8)	270 (45.7)	55 (41.4)		
	>4000	177 (24.1)	144 (24.4)	30 (22.6)		
BMI^b^	Normal	568 (77.4)	452 (76.5)	112 (84.2)	3.778	0.151
	Overweight	85 (11.6)	71 (12.0)	11 (8.3)		
	Obesity	81 (11.0)	68 (11.5)	10 (7.5)		
WC^c^	Normal	641 (87.3)	512 (86.6)	121 (91.0)	1.865	0.172
	high WC	93 (12.7)	79 (13.4)	12 (9.0)		
WHtR^d^	Normal	674 (91.8)	539 (91.2)	126 (94.7)	1.813	0.178
	Obesity	60 (8.2)	52 (8.8)	7 (5.3)		
Age	M±SD	8.64 ± 1.16	8.68 ± 1.15	8.36 ± 1.12	2.889	0.730

^a^Left-behind children: Rural registered minors under the age of 16 whose parents are both migrant workers or one is migrant workers and the other one has no guardianship ability and cannot live with their parents normally.

^b^BMI (body mass index): standards published by National Health and Family Planning Commission of the People's Republic of China in 2018 (document no.WS/T 586-−2018, Screening for overweight and obesity among school-age children and adolescents).

^c^WC (waist circumference): standards published by National Health and Family Planning Commission of the People's Republic of China in 2018 (Document no.WS/T 611-2018, High waist circumference screening threshold among children and adolescents aged 7~18 years). Girls < 7 years old were grouped according to the cut-off value of 85th percentile in each age group. Above the 85th percentile were classified as high waist circumference.

^d^WHtR (waist-to-height ratio) as central obesity screening threshold is 0.5.

### Selection and determination of trajectories model

According to the minimum absolute value of BIC and the principle of minimization of the model, the optimal trajectory numbers of BMI, WC, and WHtR of girls before pubertal onset and menarche were determined to be 3, 2, and 3, respectively, see Table 2. The AveP *P*-values of each trajectory subgroup were all higher than 0.7 suggesting that the trajectory grouping of each obesity indicator was good.

**Table 2:** The model fitting of obesity indicators before pubertal onset and menarche in girls.

**Indicators**		**Number**	**Optimal trajectory numbers**	**BIC**
Before pubertal onset	BMI	549	3	−4,094.07
	WC	549	2	−6,969.24
	WHtR	549	3	4,617.07
Before menarche	BMI	591	3	−7,514.64
	WC	591	2	−12,608.72
	WHtR	591	3	8,178.04

**Figure 2:**
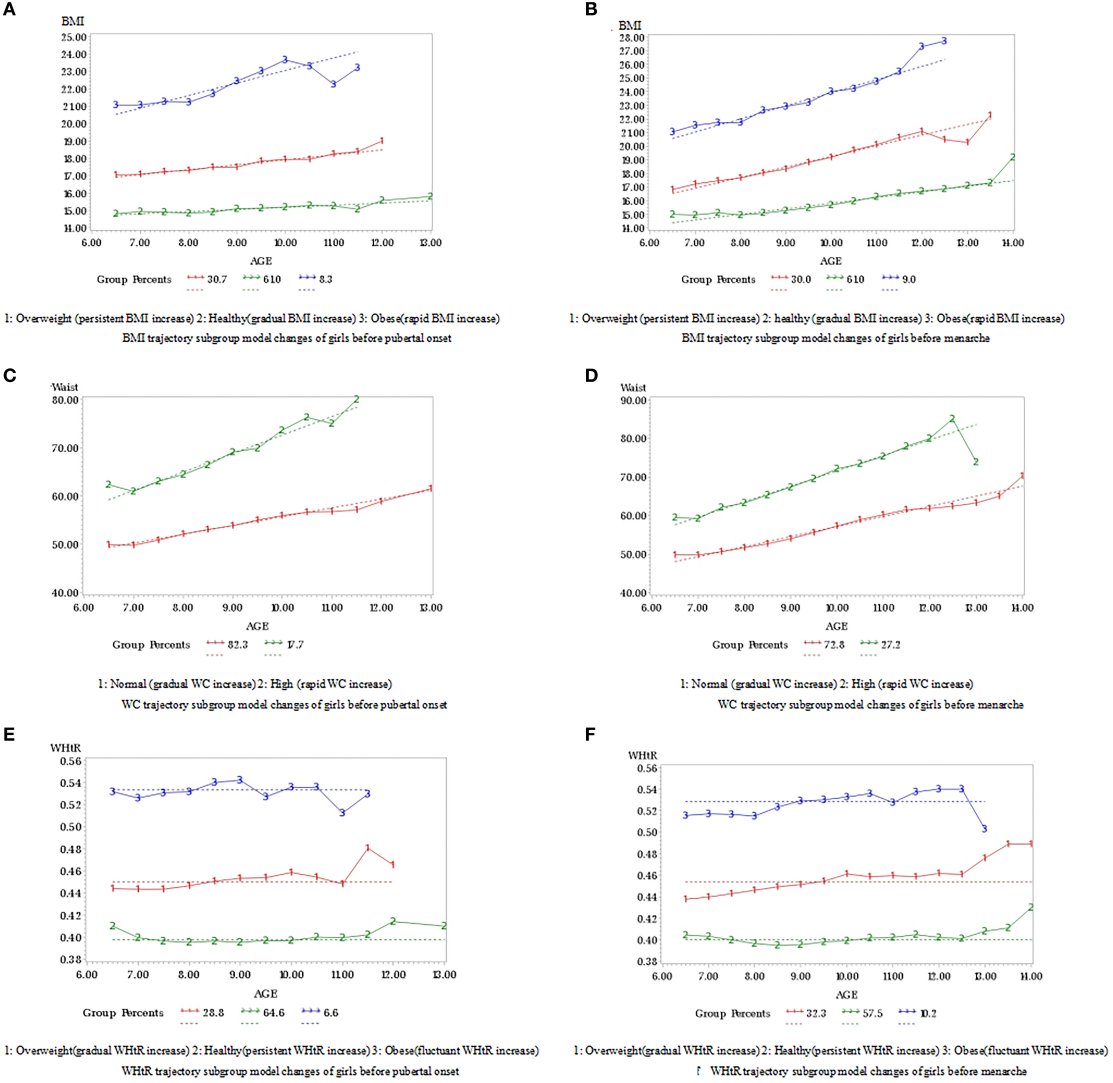
**(A–F)** Trajectory changes of BMI, waist circumference and WHtR before pubertal onset and menarche.

Based on the analysis of the trajectory model, the variation trends of each trajectory subgroup in BMI, WC, and WHtR before pubertal onset and menarche are shown in Figure 2. Girls were divided into three BMI groups (healthy, overweight, and obese), two WC groups (normal and high), and three WHtR groups (healthy, overweight, and obese). The BMI values of healthy, overweight, and obese groups were significantly different at each age stage in our study. The BMI of the healthy group incremented gradually. While the BMI of the overweight group increased persistently and that of the obese group increased rapidly. In the BMI subgroup model, the distribution in our study of healthy (gradual BMI increase), overweight (persistent BMI increase), and obese (rapid BMI increase) before pubertal onset groups were 61.0, 30.7, and 8.3%, respectively, see Figure 2A. Meanwhile, the distribution of healthy (gradual BMI increase), overweight (persistent BMI increase), and obese (rapid BMI increase) before menarche groups were 61.0%, 30.0%, and 9.0%, respectively, see Figure 2B. In the WC subgroup, girls in our study were divided into normal WC and high WC with a WC range of 50.0–60.0 and 60.0–85.0 cm, respectively. The WC in the normal group incremented gradually compared to the high group growing rapidly. The distribution of normal (gradual WC increase) and high (rapid WC increase) groups before pubertal onset was 82.3 and 17.7%, respectively, see Figure 2C. Meantime, the distribution of normal (gradual WC increase) and high (rapid WC increase) groups before menarche were 72.8 and 27.2%, respectively, see Figure 2D. The healthy, overweight, and obese WHtR groups were having WHtR values of 0.40–0.42, 0.44–0.48, and 0.50–0.55, respectively. The increment trend of the healthy group and the overweight group was persistent and gradual. However, the obese group showed a fluctuant trend. The distribution of healthy (persistent WHtR increase), overweight (gradual WHtR increase), and obese (fluctuant WHtR increase) before pubertal onset groups was 64.6, 28.8, and 6.6%, respectively, see Figure 2E. The distribution of healthy (persistent WHtR increase), overweight (gradual WHtR increase), and obese (fluctuant WHtR increase) before menarche groups was 57.5, 32.3, and 10.2%, respectively, see Figure 2F.

### Influence of the trajectory of obesity indicators before pubertal onset on the onset ages and pubertal tempo

ANOVA showed that there were statistically significant differences in each trajectory's subgroup of BMI, WC with onset age of breast, pubic hair, armpit hair development, and B2–B5 development time in girls (*p* < 0.05). Girls in the healthy (gradual BMI increase) and normal (gradual WC increase) groups had later onset age of breast, pubic hair, armpit hair development, and longer B2–B5 development time. We also observed statistically significant differences in each trajectory subgroup of WHtR with onset age of breast and pubic hair development and B2–B5 development time (*p* < 0.05). Girls in the healthy (persistent WHtR increase) group had later onset age of breast, pubic hair development, and longer B2–B5 development time, see Table 3.

**Table 3:** Comparison of pubertal development indicators under the trajectories of obesity indicators before pubertal onset.

**Indicators**	**Trajectory group**	**Onset age of breast development**		**Onset age of pubic hair development**		**Onset age of armpit hair development**		**B2-menarche development time**		**B2–B5 development time**	
		**M** ±**SD**	**t/F**	**M** ±**SD**	**t/F**	**M** ±**SD**	**t/F**	**M** ±**SD**	**t/F**	**M** ±**SD**	**t/F**
BMI	Healthy(gradual BMI increase)	10.33 ± 0.93	13.677^**^	11.86 ± 0.86	10.995^**^	12.56 ± 1.06	4.693^*^	1.86 ± 0.81	1.731	3.66 ± 0.99	41.108^**^
	Overweight (persistent BMI increase)	9.90 ± 0.90		11.50 ± 0.83		12.26 ± 0.99		1.85 ± 0.74		2.98 ± 0.82	
	Obese(rapid BMI increase)	9.94 ± 0.90		11.41 ± 0.74		12.15 ± 1.14		1.64 ± 0.82		2.37 ± 0.56	
WC	Normal (gradual WC increase)	10.25 ± 0.93	23.544^**^	11.78 ± 0.85	15.693^**^	12.51 ± 1.04	7.561^**^	1.85 ± 0.79	0.538	3.43 ± 0.98	41.331^**^
	High (rapid WC increase)	9.75 ± 0.88		11.36 ± 0.83		12.12 ± 1.09		1.79 ± 0.82		2.64 ± 0.74	
WHtR	Healthy (persistent WHtR increase)	10.32 ± 0.92	14.960^**^	11.82 ± 0.87	6.228^**^	12.53 ± 1.06	2.688	1.85 ± 0.80	1.028	3.55 ± 0.99	27.644^**^
	Overweight (gradual WHtR increase)	9.84 ± 0.90		11.53 ± 0.82		12.29 ± 1.04		1.86 ± 0.73		2.99 ± 0.86	
	Obese (fluctuant WHtR increase)	10.08 ± 0.94		11.50 ± 0.79		12.19 ± 1.10		1.66 ± 0.91		2.34 ± 0.55	

^*^p < 0.05,

^**^p < 0.01.

The multiple linear regression model showed that the initiation age of breast and pubic hair development was earlier in the overweight (persistent BMI increase) group than in the healthy (gradual BMI increase) group (B: −0.331, 95%CI: −0.515, −0.147; B: −0.341, 95%CI: −0.546, −0.136). The B2–B5 development time was shorter in the overweight (persistent BMI increase) group and the obese (rapid BMI increase) group than in the steadily healthy (gradual BMI increase) group (B: −0.568, 95%CI: −0.831, −0.305; B: −0.328, 95%CI: −0.524, −0.132), and we observed no significant differences in others (*p* > 0.05), see Table 4.

**Table 4:** The relationship between the trajectory of obesity indicators and the different onset ages of puberty and pubertal tempo before pubertal onset.

**Indicators**	**Trajectory group**	**Onset age of breast development** ^**b**^			**Onset age of pubic hair development** ^**b**^			**Onset age of armpit hair development** ^**b**^			**B2-menarche development time** ^**b**^			**B2–B5 development time** ^**b**^		
		**B**	***p***	**95%CI**	**B**	***p***	**95%CI**	**B**	***p***	**95%CI**	**B**	***p***	**95%CI**	**B**	***p***	**95%CI**
BMI^a^	Overweight (persistent BMI increase)	**−0.331**	**0.000**	**−0.515** **~** **-0.147**	**−0.341**	**0.001**	**−0.546** **~** **-0.136**	−0.257	0.075	−0.541~0.026	−0.025	0.792	−0.211~0.161	**−0.568**	**0.000**	**−0.831** **~** **-0.305**
	Obese(rapid BMI increase)	−0.121	0.136	−0.279~0.038	−0.138	0.115	−0.310~0.034	−0.097	0.406	−0.325~0.132	−0.103	0.204	−0.263~0.056	**−0.328**	**0.001**	**−0.524** **~** **-0.132**
WC^a^	High (rapid WC increase)	−0.115	0.103	−0.253~0.023	0.209	0.189	−0.103~0.521	−0.107	0.334	−0.326~0.111	0.025	0.722	−0.115~0.165	−0.007	0.945	−0.194~0.181
WHtR^a^	Overweight (gradual WHtR increase)	0.028	0.781	−0.171~0.228	0.101	0.366	−0.118~0.320	0.164	0.302	−0.148~0.476	−0.038	0.709	−0.239~0.163	−0.212	0.150	−0.500~0.077
	Obese (fluctuant WHtR increase)	0.122	0.147	−0.043~0.288	0.090	0.335	−0.093~0.274	0.040	0.741	−0.198~0.278	0.010	0.905	−0.156~0.177	−0.123	0.238	−0.328~0.082

^a^BMI, WC, and WHtR were compared with normal group.

^b^Covariates: parental divorce, parental relationship, age.

The bold values indicated that the difference was statistically significant.

### Influence of the trajectory of obesity indicators on puberty before menarche

ANOVA showed that there were statistically significant differences in each trajectory's subgroup of BMI, WC, and WHtR with age at menarche and B2–B5 development time in girls (*p* < 0.05). Girls in the healthy (gradual BMI increase) group, the normal (gradual WC increase) group, and the healthy (persistent WHtR increase) group had later age at menarche and longer B2–B5 development time, see Table 5.

**Table 5:** Comparison of pubertal development indicators under the trajectories of obesity indicators before menarche.

**Indicators**	**Trajectory group**	**Age at menarche**		**B2-menarche development time**		**B2–B5 development time**	
		**M** ±**SD**	**t/F**	**M** ±**SD**	**t/F**	**M** ±**SD**	**t/F**
BMI	Healthy (gradual BMI increase)	12.14 ± 0.95	36.066^**^	1.92 ± 0.76	0.316	3.45 ± 0.98	43.289^**^
	Overweight (persistent BMI increase)	11.53 ± 0.83		1.87 ± 0.77		2.74 ± 0.70	
	Obese (rapid BMI increase)	11.31 ± 1.15		1.93 ± 0.93		2.52 ± 0.69	
WC	Normal (gradual WC increase)	12.08 ± 0.93	65.452^**^	1.91 ± 0.75	0.033	3.35 ± 0.96	52.465^**^
	High (rapid WC increase)	11.37 ± 0.97		1.89 ± 0.85		2.68 ± 0.75	
WHtR	Healthy (persistent WHtR increase)	12.09 ± 0.97	20.038^**^	1.88 ± 0.75	0.529	3.44 ± 0.98	33.866^**^
	Overweight (gradual WHtR increase)	11.66 ± 0.85		1.95 ± 0.76		2.85 ± 0.75	
	Obese (fluctuant WHtR increase)	11.42 ± 1.23		1.90 ± 0.97		2.58 ± 0.82	

^**^p < 0.01.

The multiple linear regression model showed that the age of menarche was earlier and the B2–B5 development time was shorter in the overweight (persistent BMI increase) group than in the healthy (gradual BMI increase) group before menarche (B: −0.276, 95%CI: −0.406, −0.146; B: −0.263, 95%CI: −0.403, −0.123). Girls with high WC (rapid WC increase) had an earlier age of menarche than normal WC (gradual WC increase) (B: −0.154, 95%CI: −0.301, −0.006). The B2–B5 development time was shorter in the overweight (gradual WHtR increase) group than in the healthy (persistent WHtR increase) group (B: −0.278, 95%CI: −0.529, −0.027). There were no significant differences in others, see Table 6.

**Table 6:** The relationship between the trajectory of obesity indicators and age at menarche and pubertal tempo before menarche.

**Indicators**	**Trajectory group**	**Age at menarche** ^**b**^			**B2-menarche development time** ^**b**^			**B2–B5 development time** ^**b**^		
		**B**	**P**	**95%CI**	**B**	**P**	**95%CI**	**B**	**P**	**95%CI**
BMI^a^	Overweight (persistent BMI increase)	**−0.276**	**0.000**	**−0.406** **~** **-0.146**	0.039	0.487	−0.071~0.148	**−0.263**	**0.000**	**−0.403** **~** **-0.123**
	Obese (rapid BMI increase)	−0.109	0.144	−0.256~0.038	0.051	0.420	−0.073~0.175	−0.067	0.413	−0.227~0.093
WC^a^	High (rapid WC increase)	**−0.154**	**0.042**	**−0.301** **~** **-0.006**	−0.063	0.320	−0.187~0.061	−0.001	0.988	−0.155~0.153
WHtR^a^	Overweight (gradual WHtR increase)	0.171	0.150	−0.062~0.404	0.141	0.160	−0.055~0.337	**−0.278**	**0.030**	**−0.529** **~** **-0.027**
	Obese (fluctuant WHtR increase)	0.142	0.084	−0.019~0.304	0.029	0.672	−0.107~0.166	−0.083	0.339	−0.253~0.087

^a^BMI, WC, and WHtR were compared with normal group.

^b^Covariates: parental divorce, parental relationship, age.

The bold values indicated that the difference was statistically significant.

## Discussion

As of now, there are several studies discussing the relationship between obesity and pubertal development. Most of them used BMI as the basis for defining childhood overweight/obesity. They focused on either a single obesity indicator or simple pubertal development characteristics (9, 24). However, given the relationship between obesity and pubertal development, this study's finding is unique analyzing the influence of different trajectories of three obesity indicators on different pubertal development characteristics. To our knowledge, this is the first time to use longitudinal cohort data to explore the influence of the trajectories of BMI, WC, and WHtR on the onset ages of pubertal development characteristics and pubertal tempo among Chinese girls before pubertal onset and menarche. Our study confirms that overweight and obesity in girls can lead to earlier puberty, which is suggested in some previous studies (25–27). The main finding of this study was that prepubertal overweight and obesity can not only advance initiation ages of breast and pubic hair development in girls but also accelerate the B2–B5 development time. The B2–B5 development time was shorter in girls who were overweight (WHtR scale). The age of menarche was earlier in girls who were overweight (BMI scale) and had high WC before menarche.

Menarche is commonly used to measure girls' pubertal development. Some authors (28, 29) suggested that a higher BMI in childhood is related to an earlier age of menarche. A retrospective longitudinal cohort study in Italy (27) showed that the variation in BMI between age at thelarche and menarche and the Z-score change between body weight (BW) and BMI at thelarche has a relationship with the pubertal tempo, suggesting that early weight increase between BW and BMI at thelarche also influences the duration of puberty. Of more than 2,000 girls surveyed in the National Health and Nutrition Examination Survey (NHANES), the prevalence of menarcheal status was significantly elevated in those with BMI levels near the 85th and 95th percentile cut-off points which stands for overweight and obesity (7). In Rafique's study (29), one of the main results was the relationship between BMI and the age of menarche, the author found that 45.6% of the overweight/obese group reported early menarcheal age. The early menarcheal group had significantly higher BMI compared to the late menarcheal group. Our study indicated that girls with overweight before menarche had an earlier age of menarche and faster pubertal tempo than girls with normal BMI, which is in accordance with the former studies.

In girls, the breast tissue appearance is the initial sign of estrogenic activity, whereas the onset of pubic hair growth is due to the onset of the adrenal gland and androgen production. Researchers proposed that adipose tissue may produce aromatase, which converts androgens to estrogen and promotes puberty in girls (30). However, some scholars (27) have suggested that breast development does not necessarily herald the onset of central puberty in all cases. It may be influenced by estrogen in the adipose tissue. In our study, girls with prepubertal overweight (BMI scale) had earlier onset age of breast and pubic hair development and accelerated pubertal tempo. Similarly, girls with prepubertal obesity (BMI scale) had accelerated pubertal tempo. The risk of early menarche age and shorter B2–B5 development time in girls with overweight BMI before menarche had significantly increased. It can be seen that long-term overweight/obese status with persistent increasing BMI is more likely to cause early puberty and may affect the velocity of pubertal tempo.

The study by Fan (27) and colleagues estimated early puberty risk across BMI trajectory subgroups and investigated factors contributing to pubertal development. The authors identified four patterns of BMI trajectories applied to the latent growth mixture model (LGMM): normal BMI, rapid BMI growth, persistently overweight/obese, and early transient overweight/obese. The authors found that girls with persistently overweight/obese had the highest risk of early breast development, while girls with early transient overweight/obese had the highest risk of early menarche. In our study, we found that girls with overweight (persistent BMI increase) before pubertal onset had earlier initiation age of breast and pubic hair development as well as shorter B2–B5 development time. In addition, B2–B5 development time was shorter in girls with obesity (rapid BMI increase) before pubertal onset. We also found that girls with overweight (persistent BMI increase) before menarche had an earlier age of menarche and shorter B2–B5 development time.

Some studies have shown that WC and WHtR in children are better indicators for the distribution of body fat than BMI, easily measurable WC may be useful to help to identify children with overweight and obesity (18, 31). There was evidence that central obesity was growing faster than general obesity (assessed by BMI) among children and adolescents (32). This trend was also observed in Spanish children and adolescents aged 14 years old between 1995 and 2002 where WC measures increased, regardless of changes in BMI in both sexes. The same trend was observed in British children and adolescents, whose WC increased faster than BMI in the last 20 years (1977–1997), especially in girls (33). We found that most previous studies have used BMI as an indicator of obesity, while few studies have used WC and WHtR in pubertal development analysis. Therefore, our study analyzed the age of menarche in girls through WC and WHtR, two indicators of central obesity along with BMI to better explain the influence of obesity on menarche in girls. Our findings suggested that WC and WHtR could also serve as obesity indicators to identify the risk of earlier puberty timing and accelerated pubertal tempo. Girls with high WC (rapid WC increase) before menarche led to an earlier age of menarche and girls with overweight (gradual WHtR increase) had a shorter B2–B5 development time. Through our WC and WHtR analysis, it was found that central obesity was equally important for pubertal development in girls.

It can be seen that obesity has a great effect on the normal puberty development and pubertal tempo in girls. Meanwhile, we should take action to control obesity in girls to prevent earlier puberty and faster pubertal tempo. First of all, we should start by controlling girls' weight by guiding and encouraging them to have a healthy diet instead of overeating. Second, physical exercise and outdoor activities are good choices for teenage girls. Monitoring girls' waist circumference regularly and maintaining their weight can also effectively avoid overweight and obesity. In conclusion, in order to ensure teenage girls have healthy bodies, we need to pay attention not only to obesity but also to puberty development, and long-term trends in puberty timing and pubertal tempo. The whole society should pay attention to this topic and make plans for teenagers.

## Limitations and future directions

However, the study has some limitations. First, girls were recruited from four primary schools in an urban district of Chongqing, China, which may cause selection bias. This study only investigated the relationship between obesity and pubertal development in girls, and it is still unclear whether there is a similar conclusion on the relationship between obesity and pubertal development in boys, which suggests new research directions for us in the future. In addition, in this study, we just used covariates such as parental marital status, parental relationship, and age of children. However, we did not control confounding factors such as stressful life events, which is a limitation. In addition, our study was based on the assumption that obesity influences puberty development. However, researchers have found that the relationship might be bidirectional or pubertal development might influence obesity (4). Moreover, our study only found that obesity has an impact on pubertal development in girls but the reasons and mechanisms remain unclear. The most probable link between obesity and puberty development is leptin and its interaction with the kisspeptin system, which is an important regulator of puberty. Peripheral action of adipose tissue could also be involved in changes in the onset of puberty. Nutritional factors, epigenetics, and endocrine-disrupting chemicals are potential mediators linking pubertal onset to obesity (34, 35). Thus, future research is necessary to understand the bidirectional relationship between obesity and puberty development among children and adolescents.

## Conclusion

In summary, our findings supported that obesity has a significant influence on puberty timing and pubertal tempo in girls. We found that girls with overweight and obesity presented earlier puberty and accelerated pubertal tempo than normal girls. It is necessary to investigate the association between puberty development of adolescents and body adiposity, given the importance of fat mass quantity and distribution to health and development and the close relationship between obesity in children and puberty timing and pubertal tempo.

## Data availability statement

The original contributions presented in the study are included in the article/supplementary material, further inquiries can be directed to the corresponding author.

## Ethics statement

The studies involving human participants were reviewed and approved by the Medical Ethics Review Committee of Chongqing Medical University. The participants and participants legal guardian/next of kin provided their written informed consent to participate in this study.

## Author contributions

QL, HW, and XX participated in the design of the study. QL, XX, DW, WW, YZ, QZ, and YW were involved in data collection. QL and XX designed the analysis strategy and interpreted the results and analyzed the data. XX and DW prepared the first draft. QL and DW revised the manuscript. All authors have read and approved the final manuscript.
